# Supplementary material for: The research and development process for multiscale models of infectious disease systems
Source: PLoS Comput Biol. 2020 Apr 2;16(4):e1007734. doi: 10.1371/journal.pcbi.1007734 (PMC7156109; doi:10.1371/journal.pcbi.1007734)
Supplement: Attachment — Submitted filename: COVER_LETTER_ PCOMPBIOL.R1.pdf [file pcbi.1007734.s001.pdf]

The Editors, PLOS Computational Biology, 05 January, 2020

Dear Prof. Jennifer Flegg and Prof. Virginia Pitzer,

Thank you for giving me the opportunity to improve on my manuscript. I have now taken the opportunity to address the reviewers' comments. I also would like to thank the reviewers for taking their time to read my manuscript and making valued suggestions. My responses to reviewers are given point by point in the Table below.

No.	Comments by reviewer#1	Response Column	Page No.
Q0	This has been submitted as a research article, however it does not fit the usual format of such an article, i.e., introduction which describes a research question and context of the work, results, discussion, methods. It may be more suited to being a review (although there is limited literature cited here beyond the authors own work) or an education piece.	We agree with the reviewer that this article does not fit the usual format of a research article. Initial contacts with Plos Journal also indicated that this article does not fit the format of an education article. We cannot also classify it as review article because the articles that can be reviewed, that address multiscale modeling of disease dynamics from a general perspective in line with this article are two [1,2], and we have now added a third article [22] in the revised manuscript. However, on a balance of probabilities, we are requesting the reviewer to allow us to designate this article as a research article. This is because although the article does not fit the usual format of such an article, i.e., introduction which describes a research question and context of the work, results, discussion, methods, it does address the methods aspects for future/current problems that need to be addressed using multiscale modeling approaches.	Whole MS
Q2	It is not made clear how this framework relates to those the author has previously proposed in references [1,2]. There are a different number of levels of organisation proposed in each of these works. Why is the update /extension necessary?	In the revised manuscript we have explained how the framework in this article is related to those in [1,2,22]. In particular, we have indicated how the framework in this article is a progressive refinement of the frameworks in [1,2,22].	pp 6-7
Q3	The proposed framework	The proposed structural organization of	pp 6-

	incorporating sub-systems/levels/scales is complex. The author has provided a number of schematic diagrams to aide understanding. However, I think linking back to biological examples of the boundaries and interactions within/between these different levels of organisation would also be helpful, along with, where appropriate, citing examples in the literature of exemplar multi-scale models and their research questions.	infectious diseases consisting of sub-systems, levels, and scales is typical of the structure of a complex system, and not just infectious diseases alone. However, since we have now explained how the framework in this manuscript is a progressive refinement of the frameworks in [1,2,22], we hope that the information in these other previous articles and this manuscript will, put together, be useful in understanding the proposed framework of infectious disease systems in this manuscript incorporating sub-systems, levels, and scales.	7
Q4	It is not clear that the pathogen subsystem proposed is able to encapsulate processes of horizontal gene transfer, e.g., via phage or plasmids, which can occur between pathogen species that occupy the same niche/host and between strains of the same species during co-infection. This is especially important for the spread of antibiotic resistance in pathogen populations, and for vaccine escape, such as that which occurred for Pneumococcus (see Corander et al., 2017: https://doi.org/10.1038/s41559-017-0337-x)	We thank the reviewer for this suggestion. We have now added comments to indicate how the pathogen sub-system can be a structurally organized sub-system of an infectious disease system due to processes of horizontal gene transfer, e.g., via phage or plasmids, which can occur between pathogen species that occupy the same niche/host and between strains of the same species during co-infection.	pp 7-8, 9, 16
Q5	It is known that households provide the opportunity for prolonged close mixing between individuals and it is generally assumed that the risk of infection transmission between household contacts far exceeds that in the wider community. Also many interventions target households. Yet, households do not appear in the framework. This should at least be discussed, or incorporated into the framework.	We included the local community level, the national level, and the regional level. All the other specific cases which include households, hospitals, mass gatherings, schools, workplaces, farms, herds, etc can be addressed in the context of local level, national level, or regional level. We do not think it is necessary to include a discussion of all these specific cases.	OK
Q6	Section 2.6: Levels and scales are	We agree with the reviewer. We have	pp 23

	also linked by exchange of phage, and other mobile genetic elements, not just the pathogen, hosts, host products, etc. This should at least be discussed, or incorporated into the framework.	added a statement on this form of linkage between levels and scales of an infectious disease system in the revised manuscript.	
Q7	Related to comment 2 above: points a-e, page 22. Reference to exemplar research questions and models would aide understanding of these reasons for model selection	We have now given examples for each category of multiscales that have been developed. However, we would like the reviewer to take note that at this stage there is no variety of research questions addressed through multiscale modeling. This is mainly because, currently, where multiscale modeling approaches are used in studying disease dynamics, they tend to focus more on describing modeling approaches. The only exception is a class of IMSMs called agent based models which have been used to address a variety of research questions.	pp 26-27
Q8	Stage III. Point 3.3, page 29. There are two different, but related, types of analyses that should be conducted for any ID model: sensitivity analysis (which parameters are the outputs most sensitive too? i.e., using partial rank correlation coefficient), and uncertainty analysis (how does uncertainty in inputs affect uncertainty in outputs? i.e., using Latin Hypercube sampling of parameter space). This should be acknowledged.	Thank you for this suggestion. We have incorporated this comment in the revised manuscript.	pp 33
Q9	The author uses the terms “actual scale” and “characteristic scale” throughout, which I assume mean the same thing. It would be more clear to use one of these terms consistently, especially give there is considerable terminology being introduced in the paper around scales/levels/sub-systems.	We have now used the term “characteristic scale” throughout in the revised manuscript.	Whole MS
Q10	Point (a) on page 3. “the inability to distinguish between local	Yes. It is a multiscale modeling issue involving either a secondary multiscale	pp 18-21

	infections and imported infections in the … is because of using single scale modelling instead of multi scale modelling". Isn't this a data collection and analysis issue rather than a modelling issue? Such errors can be overcome by collected the right data, e.g., genomic data of isolates, or meta-data associated with isolates	loop or a tertiary multiscale loop/cycle. The occurance of different multiscale cycles/loops in disease dynamics (primary multiscale loop, secondary multiscale loop, tertiary multiscale loop) has now been explained in the revised manuscript in response to this question i.e. Q10 and Q16 by the same reviewer.	
Q11	Point (c) on page 5. The use of the term "local interactions" is misleading here as it suggests interactions only within a subsystem. Consider using just "interactions"	We have used term "interactions" as recommended by the reviewer in the revised manuscript.	pp 4
Q12	Figure captions should include all details necessary to understand this figure. E.g., what do (i)-(vii) refer to in Figure 1, what do arrows mean? what do (a)-(f) refer to in Figure 4	We have included all details in captions necessary to understand all the figures in the revised manuscript.	pp 9, 16, 24, 30
Q13	Page 10: throughout points (a)-(d) you use the phrase "is clear". It is not clear. Please just say what the boundary is.	We have defined the actual boundary instead of just saying the boundary "is clear" in the revised manuscript.	pp 10-11
Q14	Page 11: model categories I-V need to be described. Also provide examples from literature.	The categories are fully explained in three of our previous articles [1,2,22]. In [1] the categories are given including the categorization framework, together with several examples for each category and for each each level of organization of an infectious disease. The examples from literature are summarized in Tables for each category. In [2], we summarized the information needed to understand the five categories. In [22] (a new reference which was not in the original manuscript), we explained further these five categories in terms of the the different forms of reciprocal influence that exist between the the microscale and the macroscale in these different categories of multiscale models. We have included this additional information in the revised for	Pp 11-12

		readers who need to know more about the five different categories of multiscale models of disease dynamics.	
Q15	Use of the term “super-infection” throughout. When this term is first used it should be defined, as there is not consensus in literature. It is used to mean both strain replacement (superseding infection) as well as co-infection (co-existence) of strains within a host.	We have included the definition that “super-infection” in this article means “repeated infection by pathogen of the same species/strain before the host recovers from prior infection by the same species/strain” in the revised manuscript.	pp 16, 17
Q16	Page 17: Is it not true that global exchange of organisms can also play a role in disease persistence? E.g, pathogen moving to population with higher levels of susceptibility, and being re-introduced after a period of time, or TB bacteria moving from airways to granuloma where it can persist for long periods of time?	Yes, there can a multiscale cycle involving the reciprocal influence persistence of infection and dispersal/spread of infection. This multiscale cycle/loop can be either a secondary multiscale loop/cycle or a tertiary multiscale loop/cycle depending on the scale of multiscale observation used in the development of the multiscale model. In the revised manuscript, we have now explained the occurance of different multiscale cycles/loops in disease dynamics (primary multiscale loop, secondary multiscale loop, tertiary multiscale loop) in response to this question and Q10 by the same reviewer.	pp 18-21
Q17	Point e, page 31. How do mass drug administrations fit in here? They are treatments, but given to people not necessarily infected.	Mass drug administration falls under public health interventions level/scale in the first category of public health interventions: (i) clinical prevention interventions - which are offered by health-care workers and usually in clinical settings and involve using some drugs (e.g. mass drug administration) or vaccines. We have included this additional information in the revised manuscript.	Pp 35
**No.**	**Comments by reviewer#2**		
Q0	The authors make good case for multiscale modeling of infectious	Thank you	OK

	diseases from a complex systems perspective. The paper provides developmental process maps for multiscale modeling systems, in particular the integration of four different multiscaling approaches to address the infectious disease dynamics is interesting.		
Q1	In the vast literature of epidemic modeling examples of each aspect of multiscale nodeling system proposed by the author.	We have now given examples for model selection for each of the five categories of multiscale models.	pp 26-27
Q2	correlating the existing studies for a well studied epidemic (e.g. AIDS, H1N1, Ebola etc) into the hierarchical format suggested by the author in figure 2 would help identify where the specialized studies fit in a complex systems approach.	This is OK. But we think that this will make the manuscript longer than originally intended. We will however, address this aspect in a different manuscript. We also think that the explanation given in the revised manuscript is now detailed enough for anyone to carry out this task.	OK
Q3	The same comment for the integration of multiscale modeling approaches in figure 4. It would be helpful to identify examples of past research that fits into each category. This would be needed to bring out the interplay between different modeling approaches for the suggested integrated system.	In the original manuscript, we gave examples of E-MSMs as experimental systems, culture systems, clinical trial systems, observational systems or surveillance systems. We do not it is necessary to give an example of a specific clinical trial or a particular experimental system as an example of E-MSM. We also gave a range of examples for M-MSMs and D-MSMs under five categories of multiscale models. We also indicated any computer program to solve a M-MSM or D-MSM is itself a C-MSM. We do not think we should give an example of a computer program that solves a M-MSM or D-MSM. We have however included an example of a study that investigated the interplay between M-MSMs and D-MSMs (see ref. [24] in revised MS).	pp 32

Thank you,
Winston Garira, PhD
